# Consequences of chronic diseases and other limitations associated with old age – a scoping review

**DOI:** 10.1186/s12889-019-7762-5

**Published:** 2019-11-01

**Authors:** Petra Maresova, Ehsan Javanmardi, Sabina Barakovic, Jasmina Barakovic Husic, Signe Tomsone, Ondrej Krejcar, Kamil Kuca

**Affiliations:** 1 Department of Economics, Faculty of Informatics and Management, University of Hradec Kralove, Rokitanskeho 62, 500 03 Hradec Kralove, Czech Republic; 2 Faculty of Traffic and Communications, University of Sarajevo, Sarajevo, Bosnia and Herzegovina; 3 Faculty of Electrical Engineering, University of Sarajevo, Sarajevo, Bosnia and Herzegovina; 4 Faculty of Rehabilitation, Riga Stradinš University, Riga, Latvia; 5 Center of Basic and Applied Research, Faculty of Informatics and Management, University of Hradec Kralove, Rokitanskeho 62, 500 03 Hradec Kralove, Czech Republic; 6 Malaysia Japan International Institute of Technology (MJIIT), Universiti Teknologi Malaysia Kuala Lumpur, Jalan Sultan Yahya Petra, 54100 Kuala Lumpur, Malaysia

**Keywords:** Chronic diseases, Seniors’ needs, Elderly disability, activities of daily living

## Abstract

**Background:**

The phenomenon of the increasing number of ageing people in the world is arguably the most significant economic, health and social challenge that we face today. Additionally, one of the major epidemiologic trends of current times is the increase in chronic and degenerative diseases. This paper tries to deliver a more up to date overview of chronic diseases and other limitations associated with old age and provide a more detailed outlook on the research that has gone into this field.

**Methods:**

First, challenges for seniors, including chronic diseases and other limitations associated with old age, are specified. Second, a review of seniors’ needs and concerns is performed. Finally, solutions that can improve seniors’ quality of life are discussed. Publications obtained from the following databases are used in this scoping review: Web of Science, PubMed, and Science Direct. Four independent reviewers screened the identified records and selected relevant publications published from 2010 to 2017. A total of 1916 publications were selected. In all, 52 papers were selected based on abstract content. For further processing, 21 full papers were screened.”

**Results:**

The results indicate disabilities as a major problem associated with seniors’ activities of daily living dependence. We founded seven categories of different conditions - psychological problems, difficulties in mobility, poor cognitive function, falls and incidents, wounds and injuries, undernutrition, and communication problems. In order to minimize ageing consequences, some areas require more attention, such as education and training; technological tools; government support and welfare systems; early diagnosis of undernutrition, cognitive impairment, and other diseases; communication solutions; mobility solutions; and social contributions.

**Conclusions:**

This scoping review supports the view on chronic diseases in old age as a complex issue. To prevent the consequences of chronic diseases and other limitations associated with old age related problems demands multicomponent interventions. Early recognition of problems leading to disability and activities of daily living (ADL) dependence should be one of essential components of such interventions.

## Introduction

The phenomenon of the increasing ageing population is one of the most important economic, social, and medical issues of current times. Recent demographic trends outline that the number of people of old age will continue to rise dramatically. Today most people can expect to live to age 60 and beyond [1]. Between 2000 and 2020, the fastest growing segment of the United States (US) population will be individuals aged 65 years and older [2]. By 2030, the number of people in the world aged 60 years or over will increase by 56%, and by 2050, the global population of senior persons is projected to more than double its size in 2015. The number of people aged 65 or older is about to grow to nearly 1.5 billion in 2050, with most of the increase in developing countries [3]. These demographic transitions essentially require shifting the global focus to cater for the preventive healthcare and medical needs of the elderly population [4]. A wide gamut of determinants, such as social concerns and maltreatment of elderly individuals, poor knowledge and awareness about the risk factors, food and nutritional requirements, psycho-emotional concerns, financial constraints, health care system factors, and physical correlates determine the medical problems and thus cast a significant impact on the quality of life (QoL) of elderly individuals [5–8].

The ageing population tends to have a higher prevalence of chronic diseases worldwide today [9]. For example, six in ten adults in the US have a chronic disease and four in ten adults have two or more [10], while Sweden reports multiple chronic conditions of 56.3% [11]. These chronic conditions are significant and profound economic issue for any person, the healthcare system, and society as a whole [12]. Such diseases account for 86% of all medical costs in the US being even greater worldwide [13]. The presence of multiple chronic conditions in the same individual has profound implications for healthcare costs and utilisation [14]. For example, a Swiss study estimated that the average total healthcare costs were 5.5 times higher in elderly patients with multiple chronic conditions as compared with elderly patients without multiple chronic conditions [15]. The combined cost savings from the health and productivity that results from a small reduction in the prevalence of chronic disease cannot be ignored, resulting in a genuine return on investment in a very small span of time [16].

Chronic diseases require a long period of treatment that leads to the increase in demand for healthcare services and changes its nature [17–21]. This need for long-term care can lead to a decline in the QoL of elderly individuals [22]. This phenomenon will put pressure on healthcare systems to adapt in order to meet these changing demands. New and emerging technologies have the potential to change healthcare at home and in community [23]. Recognising the needs of elderly individuals suffering from chronic diseases and other constraints would fix many problems that patients face and results in an improved QoL, safety and overall health. Also, by investing in better QoL, safety and overall health in elderly, their productivity will rise as well thereby contributing to the economic and social opportunities.

This paper will be presenting an up-to-date survey of the limitations of seniors in connection with their chronic diseases and helps to provide a more detailed image of the research that has been done in this field. Additionally, the paper underlines the main research areas within seniors’ needs in relation to chronic diseases and the limitations associated as an informative summary for further research. To appreciate the scientific progress that has been made, with an emphasis on the literature dedicated to the economic field, a systematic review has been conducted by using keywords such as elderly activities of daily living (ADLs), elderly QoL, and elderly instrumental activities of daily living (IADLs).

This paper is organized as follows: After the Introduction covering the background for this research given in the first section, section 2 describes in detail the used methodology. The challenges faced by seniors with chronic diseases and other limitations associated with old age are specified in section 3. After reviewing seniors’ needs and concerns, the solutions that can improve their QoL are discussed in section 4. Finally, section 5 concludes the paper and gives open research areas for the future activities.

## Methods

This scoping review is performed to identify and summarise up-to-date conditions leading to ADLs dependency in relation to chronic diseases and other limitations associated with old age. “A 4-step systematic review was conducted using empirical studies: locating and identifying relevant articles; screening located articles; examining full-text articles for inclusion or exclusion; and a detailed examination of the 21 articles included.”

### Search strategy and eligibility criteria

In February 2018, four investigators performed a systematic literature search of the Web of Science, PubMed, and Science Direct. The period of interest covered the years from 2010 to 2017, and the electronic search included the following keywords: elderly and (Activities of daily living) ADLs, elderly QoL, elderly and (Instrumental Activities of daily living) IADLs, senior and ADLs, and senior and ADLs.

“In the Web of Science database, 829 publications were identified. Only two types of publications were considered eligible for the purpose of this study: ‘article’ and ‘review’, which includes the following: ‘review articles’, ‘research articles’, ‘data articles’, ‘book reviews’, ‘mini reviews’, ‘product reviews’, and ‘video articles’. This criterion reduced the initial set of papers to a total of 172 research articles, which were selected for further processing. For PubMed and Science Direct, a semi-automated framework or aiding surveys was used [24]. The framework first used the search tools of the libraries with the aforementioned keywords. Next, after eliminating duplicate records from the retrieved papers, it analysed the title, abstract, and keywords section of each paper to evaluate whether any of the following properties or their synonyms (listed in parenthesis) were mentioned: ADLs and IADLs. The paper distribution based on property (keyword) is presented in Table 1.”

**Table 1:** Paper distribution by property

Key words (used ‘AND’ between all words)	WOS	Science Direct	PubMed
ADLs (activities of daily living)	107	23	109
Elderly ADLs	1	3	14
Elderly quality of life	697	129	780
IADLs (instrumental activities of daily living)	22	4	24
Elderly IADLs	0	0	0
Senior ADLs	1	1	0
Senior IADLs	1	0	0
Total	829	160	927

ADLs (or ADL) is a term used in healthcare to refer to people’s daily self-care activities. Common ADLs include feeding, bathing, dressing, grooming, working, homemaking, cleaning after defecating, and leisure [25]. Adaptive equipment and devices may be used to enhance and increase independence in performing ADLs. Basic ADLs consist of self-care tasks that include but are not limited to, bathing and showering, personal hygiene and grooming, dressing, toilet hygiene, functional mobility, and self-feeding. One way to think about basic ADLs is that they are the things many people do when they get up in the morning and get ready to go out of the house: get out of bed, go to the toilet, bathe, dress, groom, and eat [26]. IADLs, such as cleaning and maintaining the house, preparing meals, shopping, managing money, moving within the community and many other activities are not necessary for fundamental functioning, but they let an individual live independently in a community [27].”

This paper includes 1916 publications that were acquired based on a given set of properties from different databases. The publications were automatically analysed and assessed, and then four properties were chosen to be further processed from the existing pool. False positive papers that had the relevant properties but were not relevant to the study at hand were discarded manually after being checked. This resulted in the overall pool of studies being narrowed down to 52 papers and articles. This set was then processed further, and the final number was brought down to 21 full length papers subsequent to the manual and semi-auto search. This excluded papers that did not fall into the description underscored in subsection 2.2.

### Data extraction and study quality evaluation

Each publication’s data was extracted – the main team consisted of four researchers that worked to outline data individually. The following data was extracted: the country and type of study, the study’s author, and the study’s title. A study was included if it qualified as per the following requirements:

- Published after 2010;
- Focused on chronic diseases and other limitations associated with old age;
- Posed questions concerning seniors’ needs;
- Described diseases or specific needs of seniors;
- Discussed the limitations of seniors in connection with their chronic diseases;
- Focused on elderly individuals’ QoL and ADLs;
- Focused on older people’s community-dwelling; and
- It was in English.

A publication was not included if any of the following were true:

- It was not in English;
- It included a theoretical model;
- Focused on a technical description of the solution;
- Described systems for the diagnosis of the disease;
- Described healthcare management systems; and
- Focused on a disease rather than focusing on the patient.

**Fig. 1:**
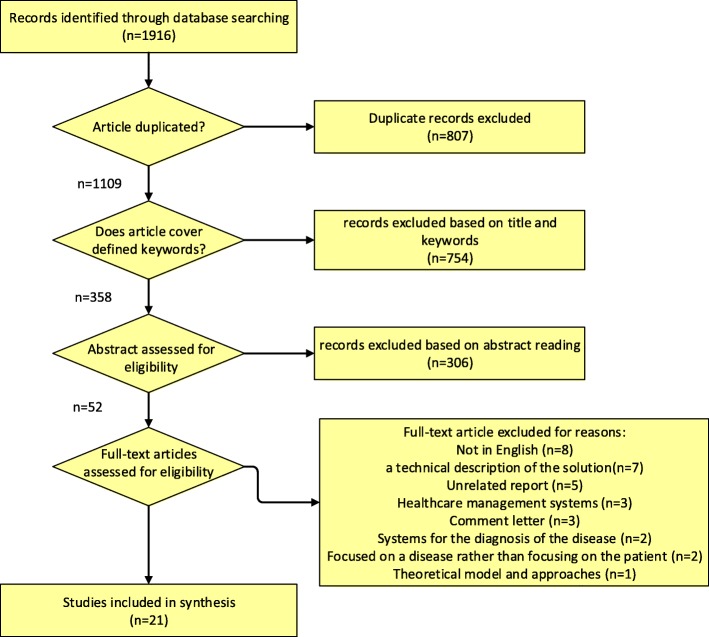
Publication search process according to PRISMA

Search process is described in Fig. 1.

## Results

Current research on ageing problems and seniors’ ADLs dependency is described in Table 2. For each study, the objectives, problems and diseases, main findings, and limitations are described. Elderly persons will, as they age, continue to progressively decline in terms of their functional capacity. This will affect their frailty, worsen dependency and add to their loss of autonomy [48]. Ageing results in considerable and consistent change in an organism and results in a decline of or limited physical function and an augmented level of comorbidity [40].

**Table 2:** Summary of studies

Authors (Year)	Title	Objective	Problems and diseases	Sample	Methods	Results	Limitations	Comments
Velázquez Alva et al. (2013) [28]	“The link shared by sarcopenia, physical mobility, undernutrition, and basic activities of daily living in the context of older women from Mexico City”	assessing the association between sarcopenia and mobility, and basic activities of daily living (ADLs) of elderly women	Sarcopenia	90 women in Mexico City	“Baumgartner’s equation, The SENECA questionnaire, Katz index, linear regression models”	“Patients with sarcopenia had a higher prevalence of undernutrition and difficulty in climbing stairs -In terms of ADL, 64.9% of patients had intermediate independence.”	“cross-sectional design that doesn’t allow identifying causal links. Invalid Baumgartner’s equation”	“Sarcopenia is associated with difficulties in mobility and a higher prevalence of undernutrition, particularly difficulties in climbing stairs.”
Haewon Byeon et al. (2016) [29]	“The relationship between communication activities of daily living and quality of life among the elderly suffering from stroke”	investigate the relationship between (C-ADL^1^) and quality of life of elderly stroke patients to enhance the quality of life (QoL)	stroke	165 elderly, with stroke	“using multiple regression analysis between C-ADL and SSQOL^3^”	“C-ADL had a significant positive relationship with SSQOL. It is necessary to enhance stroke patients’ communication ability in daily living to raise their QoL.”	“exclusion of patients with severe aphasia, for whom test could not be conducted.”	“Some activity to enhance stroke patients’ communication ability in daily living to raise their QoL.”
Charernboon, T. et al. (2016) [30]	“Characteristic Profiles of Activities of Daily Living and Relationship with Cognitive Performance in Thai Elderly with Different Stages from Normal Cognitive Function, Mild Cognitive Impairment to Dementia”	“evaluating the functional difference among normal cognitive elderly, (MCI^2^), and dementia; also, the relationship between cognitive performance and functional abilities”	dementia	90 participants	“Spearman’s rank-order correlation, the Mann–Whitney U-test, Multiple regression model”	“IADL can be subtly impaired in people with MCI but markedly impaired in those with mild dementia. BADL^1^ begins to decline in moderate dementia and then reaches a level of severe impairment in severe dementia.”	demographic characteristics and cultures that are similar	“Designing specific ADL assessment tools for MCI would help in better differential diagnosis and prognosis for patients.”
Chen Shen, et al. (2018) [31]	“Unmet needs of activities of daily living among a community-based sample of disabled elderly people in Eastern China: a cross-sectional study”	“identify the prevalence and risk factors of unmet needs among the disabled elderly in China”	disabled elderly	303 older adults in China	“Using the Barthel Index (BI) and Functional Activities Questionnaire (FAQ), binary logistic regression analysis”	“93.1% of the disabled people had at least one unmet need. The unmet needs were using vehicles, stairs, working on a hobby social interaction and ambulating. The factors influencing unmet needs were related to the degree of disability in (IADL), the relationship with caregivers and the monthly income of caregivers.”	“collect more samples from different regions, find a better way to improve the precision and multiplicity of analyses”	“government and caregivers should take more action to prevent or reduce unmet needs among elderly individuals.”
Costa, FA, et al. (2018) [32]	“Contribution of chronic diseases to the prevalence of disability in basic and instrumental activities of daily living in elderly Brazilians: the National Health Survey”	“assess the contribution of selected chronic diseases to the prevalence of disability in elderly”	chronic diseases	10,537 elderly Brazilians	“A multinomial additive hazards model assesses the contribution”	“Contribution of chronic diseases to prevalence of disability was greater in younger elderly and highlighting the relevance of stroke and arthritis in men, and arthritis, hypertension, and diabetes in women.”	“a cross-sectional design, which does not allow for establishment of a temporal relationship”	“orient health services to target specific groups, considering age, sex, and current illnesses, aimed at preventing disability in elderly individuals.”
Farías-Antúsnez et al. (2018) [33]	“Disability related to basic and instrumental activities of daily living: a population-based study with elderly in Pelotas, Rio Grande do Sul”	“estimate the prevalence of disability related to basic and instrumental activities of daily living”	Disability	1.451 elderly in Brazil	“Poisson regression”	“Functional disability was associated with individuals older than 80 with less schooling years and affected by multiple morbidities.”	“possible reverse causality bias in some associations”	“social policies need to adjust to the new reality with the elaboration of policies and programmes focused on the health of elderly individuals.”
Furuta, M, et al. (2013) [34]	“Interrelationship of oral health status, swallowing function, nutritional status, and cognitive ability with activities of daily living in Japanese elderly people receiving home care services due to physical disabilities”	“examining direct and indirect relationships among oral health status, swallowing function, nutritional status, cognitive ability, and ADL in elderly”	physical disabilities	“286 elderly people living at home and receiving home care services”	“the BI, the Clinical Dementia Rating Scale, Path analysis to test pathways”	“Poor oral health status and cognitive impairment had a direct effect on denture wearing, and the consequent dysphagia, in addition to cognitive impairment, was positively associated with malnutrition.”	“a longitudinal study is needed to examine a temporal relationship.”	“maintaining or improving oral health status and swallowing function indirectly or directly contribute to preventing a decline in ADL in elderly people who require home care.”
Genkai, S.et al. (2015) [35]	“Loss of occlusal support affects the decline in activities of daily living in elderly people receiving home care”	“clarifying whether the absence of occlusal support would lead to a decline in ADL in elderly people receiving home care”	occlusal support	322 elderly in four prefectures	the Barthel Index	“““Factors related to declines in ADL in elderly people receiving home care were cognitive function and occlusal support.”	“small size of samples”	“the absence of occlusal support was a significant risk factor for a decline of the ADL in elderly people receiving home care.”
Ha, E; Kim, K (2013) [36]	“Factors that influence activities of daily living in the elderly with probable dementia”	“Identifying the factors that influence ADL in elderly individuals with probable dementia”	dementia	152 elderly in South Korea	Using independent t-tests, Pearson’s correlation and stepwise multiple regression	“Factors affecting ADL in elderly individuals with probable dementia were faecal incontinence, regularity of exercise, cognitive function, urinary incontinence, and CVA history.”	“further refinement of the underlying model is warranted.”	“Multidisciplinary interventions are essential to improve the ADL and prevent deterioration of cognitive function in elderly patients with probable dementia.”
Hesseberg, K.et al. (2013) [37]	“Disability in Instrumental Activities of Daily Living in Elderly Patients with Mild Cognitive Impairment and Alzheimer‘s Disease”	“The aim is to examine disability in (IADL) in elderly persons”	Mild Cognitive Impairment and Alzheimer‘s Disease	729 patients	Multiple logistic regression	“Found an association between IADL and diagnosis, and a difference in the proportion of disability in IADL in patients with MCI and AD.”	“cross-sectional design that does not allow to conclusion on causality.”	“Problems with handling medication, shopping and preparing food are domains clinicians should be aware of regarding IADL disability.”
Hou, C, et al. (2018) [38]	“Trends of activities of daily living disability situation and association with chronic conditions among elderly aged 80 years and over In China”	“Investigating the cross-sectional trends of prevalence and severity of ADLs in old people and identifying the potential risk factors of disability”	Stroke and cognitive impairment	52,667 participants in China	GEE^5^ models with a logistic link and binominal distribution	“Prevalence of ADL disability declined among the old population without obeying a linear pattern. Temporal trends of ADL disability mainly attributed to the change of low disability level prevalence. Stroke/CVD and cognitive impairment were the most common risk factors of disability. Vision impairment-caused disability has become less common.”	“association of some important chronic diseases such as arthritis and depression symptom, and the effects of severity of diseases on functional disability were not examined”	“these findings from this study could provide information to develop preventive strategies and specific interventions for the reduction of disability in the oldest Chinese population.”
Masoudi Alavi N, et al. (2014) [39]	“Dependency in Activities of Daily Living Following Limb Trauma in Elderly Referred to Shahid Beheshti Hospital, Kashan, Iran in 2013″	“evaluating elderly independence in ADLs following limb trauma and its related factors in patients”	Limb Trauma	200 traumatic patients in Iran	Chi-square test, One-way and two-factor ANOVA, and Multiple regression analysis.	“More than three-quarters of elderly individuals were independent in ISADL before the trauma, but trauma in elderly patients had a substantial negative effect on patients’ ability and ADL function.”	“ISADL questionnaires were completed by phone call one to three months after trauma, which might affect the responses.”	“recommend continuing patients’ follow-up for longer periods and interventional studies to improve ISADL following trauma in elderly.”
Okabe, T, et al. (2017) [40]	“Age-specific risk factors for incident disability in activities of daily living among middle-aged and elderly community-dwelling Japanese women during an 8–9-year follow-up”	“investigating risk factors for incident disability in ADL and determining whether there are differences in risk factors according to age groups”	incident disability	264 Japanese women	Student’s t-test used for continuous variables, the χ2-test, and regression analysis.	“A different set of risk factors was associated with incident ADL disability among women aged 40–64 years and women aged ≥65 years.”	“included only women, these results cannot be generalised to men.”	“Age-specific screening and intervention strategies are necessary for effective prevention of incident ADL disability.”
Orive, M; et al. (2015) [41]	“Changes in health-related quality of life and activities of daily living after hip fracture because of a fall in elderly patients: a prospective cohort study”	“evaluating changes in HRQoL^e^ and the ability to conduct ADL among patients with hip fracture because of a fall”	hip fracture	150 Adults aged 65 or older	the BI and the Lawton Brody Index for ADL, the non-parametric Wilcoxon test	“Hip fractures have profound effects on HRQoL and ADL in both men and women, regardless of age.”	small sample	“The need for special follow-up care of elderly hip fracture patients in the immediate and late post fracture periods.”
Quail, JM, et al. (2011) [42]	“Unmet Need for Assistance to Perform Activities of Daily Living and Psychological Distress in Community-Dwelling Elderly Women”	“examining the possible association of physical assistance need with psychological distress”	psychological distress	530 women in Canada	multivariable linear regression	“Receiving assistance to meet IADL needs is associated with elevated psychological distress. Not receiving assistance, however, is associated with even greater distress.”	relied on self-reported disability, which may have caused some misclassification of disability.	“It is essential to provide elderly persons with the support they need and will accept to adapt both physically and mentally to declining health and function.”
Safa, A, et al. (2016) [39]	“Predictive Factors of Dependency in Activities of Daily Living Following Limb Trauma in the Elderly”	“evaluating the predictive factors of dependency in ADL following limb trauma in elderly”	Limb Trauma	200 patients, in Iran	the t-test and analysis of variance (ANOVA) and the multiple regression analysis	“Many factors, such as gender, age, education, type of trauma, and location of the injured organ, may predict ADL following limb trauma.”	small sample	“Knowing the predictive factors of dependence in ADL after trauma may help health systems to design effective and realistic strategies for rehabilitative programmes.”
Silva, AD, et al. (2014) [43]	“Association between the degree of physical impairment from leprosy and dependence in activities of daily living among the elderly in a health unit in the State of Minas Gerais”	“determining whether physical impairment from leprosy is associated with dependence among elderly individuals”	leprosy	186 elderly persons	the Statistical Package for the Social Sciences (SPSS)	“Leprosy physical impairment grade is associated with dependence for IADL, creating the need for greater social support and systematic monitoring by a multidisciplinary team.”	uses secondary data	“The importance of early diagnosis and treatment of leprosy to prevent physical impairment and dependence in later years.”
Takemasa S, et al. (2017) [44]	“Interrelationship among the health-related and subjective quality of life, daily life activities, instrumental activities of daily living of community-dwelling elderly females in orthopaedic outpatients”	““examining the health-related and subjective QoL of community-dwelling elderly females in orthopaedic outpatients, and how such QoL correlates with their ADL and IADL”	orthopaedic	27 elderly females	Using Spearman’s rank	“For community-dwelling elderly females to maintain good subjective and health-related QoL, controlling their pain is critical so that excruciating pain does not restrict their activities. They also need to maintain good health, feel happy and comfortable and have more opportunities for social interaction.”	small sample. Did not include men in the study	“supporting community-dwelling elderly females in orthopaedic outpatients to improve their sense of physical and mental well-being, and prevent and reduce their depression and physical pain, is required to improve their QoL.”
Liu, Y, et al. (2012) [45]	“The Unmet Activities of Daily Living (ADL) Needs of Dependent Elders and their Related Factors: An Approach from Both an Individual- and Area-Level Perspective”	“examining disabled elders’ unmet ADL needs and the factors associated with those unmet needs”	disabled elders	6820 elders from Taiwan	hierarchical linear modelling	“Highest percent of unmet ADL needs was for climbing stairs. The following factors as important: education level; living arrangements; number of illnesses; number of IADL limitations; caregiver’s age; the caregiver–patient relationship; care burden; household size.”	majority of respondents were female	“social welfare expenditure moderates unmet needs by provision of services, which means that the positive effect of social welfare services cannot be ignored; the government should play a supervisory role.”
Putthinoi, S. et al. (2016) [46]	“Performance in Daily Living Activities of the Elderly While Living at Home or Being Home-bound in a Thai Suburban Community”	“examining the ability of elderly in performing their daily activities while living at home or being home-bound in the community”	caducity	32 home-bound elderly people	cross-sectional survey	“Elderly individuals communicated independently. They acted independently in interpersonal interactions and relationships, but they needed assistance from people or equipment in some activities. Most of the home-bound elderly performed ADL independently, whereas elderly individuals living at home were dependent when using transportation and driving.”	small sample size	“Individuals living with chronic health conditions, most subjects could not independently perform these activities; transportation community and, social and civic life.”
Ohri, P. et al. (2014) [47]	“A Study of Daily Living Dependency Status among Elderly in an Urban Slum area of Dehradun”	“assessing the daily living dependency status among elderly individuals”	caducity	215 elderly	A cross-sectional study	“Out of the total, 93% individuals were independent in their ADLs. Maximum inability was found in bathing and dressing. In total, 70.7% of elderly were dependent on one or more IADLs, female elderly being more dependent than males.”	other parameters of geriatric age could be evaluated, such as nutritional status and morbidities in elderly	“Education and socioeconomic status had a positive effect on independence. Male elderly showed a maximum dependency for cooking and laundry, while females showed a greater dependency in using the telephone, managing money, and travelling.”

^1^Communication Activities of Daily Living

^2^Stroke-Specific Quality of Life

^3^Mild cognitive impairment

^4^Basic activities of daily living

^5^Generalized estimating equation

^6^Health-related quality of life

Identified major problems that cause seniors’ ADL dependency are classified as follows:

- Disabilities and unmet needs are mentioned in 13 articles.
- Psychological problems are mentioned in six articles.
- Difficulties in mobility are mentioned in four articles.
- Poor insight and cognitive function are mentioned in four articles.
- Falls and incidents are mentioned in four articles.
- Wounds and injuries are mentioned in three articles.
- Prevalence of undernutrition and dysphagia are mentioned in three articles.
- Communication problems are mentioned in one article.

**Fig. 2:**
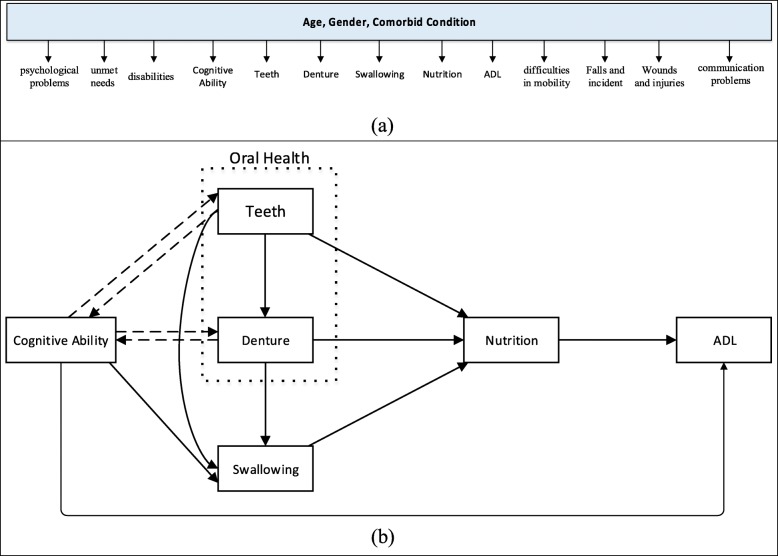
(a) Age, gender, and comorbid condition are factors that affect seniors’ conditions. (b) Part of the potential causal pathway by which problems directly or indirectly affect ADL in elderly people. ADL in elderly individuals is impaired by several factors [34]

The prevalence and the number of comorbidities increase with age, which might lead to ADL dependency [40]. Part of the potential causal pathway by which the aforementioned problems directly or indirectly affect ADL in elderly people is presented in Fig. 2(a) and (b) [34]. In Fig. 2(a), the findings of the selected studies revealed that as a person ages, his/her psychological issues are intensified and s/he feels more unmet needs [31–33, 36, 44]. Furthermore, aging is a factor that could increase the range of disabilities, reduce cognitive abilities, and increase problems related to the teeth, swallowing and nutrition [30, 34] –[42]. Along with these issues, as research has shown, older people find it more difficult to perform everyday activities, move around, and communicate with others; meanwhile they are more likely to fall down, experience incidents, and suffer from wounds and different types of injuries [29, 39, 41]. In Fig. 2(b), there is a conceptual model showing the mutual effects of some aging-related problems on activities of daily living (ADLs). As this dynamic cause-effect diagram illustrates, reduced cognitive abilities and oral hygiene mutually affect each other. Problems could lead to malnutrition in the elderly. The swallowing function, cognitive abilities, and the quality of nutrition can directly affect ADLs.

Table 3 shows the various problems and their impacts on the seniors’ ADL on the basis of selected articles.

**Table 3:** Problems of elderly people and the articles that refer to them

Authors (Year)	Disabilities and unmet needs	Psychological problems	Difficulties in mobility	Poor insight and cognitive function	Falls and incident	Prevalence of undernutrition and dysphagia	Wounds and injuries	Communication problems
Velázquez Alva et al. (2013) [28]			*			*		
Haewon Byeon et al. (2016) [29]	*	*						*
Charernboon, T. et al. (2016) [30]				*				
Chen Shen, et al. (2018) [31]	*	*						
Costa, FA, et al. (2018) [32]	*	*						
Farías-Antúşnez et al. (2018) [33]	*							
Furuta, M, et al. (2013) [34]	*			*		*		
Genkai, S.et al. (2015) [35]			*			*		
Ha, E; Kim, K (2013) [36]		*		*				
Hesseberg, K.et al. (2013) [37]				*				
Hou, C, et al. (2018) [38]	*							
Masoudi Alavi N, et al. (2014) [39]	*		*		*		*	
Okabe, T, et al. (2017) [40]	*		*		*			
Orive, M; et al. (2015) [41]					*			
Quail, JM, et al. (2011) [42]	*	*						
Safa, A, et al. (2016) [39]	*				*		*	
Silva, AD, et al. (2014) [43]							*	
Takemasa S, et al. (2017) [44]		*						
Liu, Y, et al. (2012) [45]	*							
Putthinoi, S. et al. (2016) [46]	*							
Ohri, P. et al. (2014) [47]	*							

### Disabilities and unmet needs

Disability is one of the most common problems in seniors that leads to ADL dependence; dependence in ADL and IADL is a critical “challenge for community-dwelling elderly people, regardless of whether their needs are met or unmet. As the elderly population continues to grow, the challenges involved in addressing disability and unmet need will also grow [42]. Frailty describes the condition of elderly persons with the highest risk of disability, institutionalisation, hospitalisation, and death [49]. Chronic conditions have been confirmed as the main causes of disability [38].”

“Diseases show a greater contribution to the prevalence of more severe disability, that is, with impairment of basic ADL. Elderly patients with a diagnosis of arthritis, stroke, or diabetes should be monitored more effectively by considering the important contribution of these conditions to disability. Stroke and arthritis were the diseases that contributed most consistently to disability, independent of sex and age bracket. Hypertension and heart disease showed only a significant contribution to the prevalence of both levels of disability in women [32, 33]. Disabling effects of multimorbidity increased in ADL dependency [38].”

“Disabilities after stroke become chronic, and the inability to independently perform ADLs, such as dressing and eating, in a long run causes helplessness and depression in stroke patients and inflicts emotional pain, such as intellectual regression, despair, and anxiety. Functional disorders in daily life in long term are likely to cause deterioration in QoL of stroke patients and maladjustment in social relationships, changes in role, and economic difficulties [30].

“Having knee joint or back pain was significantly associated with a higher risk of incidence of ADL dependency. Older adults with pain have a higher risk of developing incident ADL dependency and commonly have functional limitations. Speculation has indicated that in mutual feedback loops in which pain and functional limitations are mutually reinforcing, pain exacerbates functional limitations and functional limitations exacerbate pain. Age-specific screening and intervention strategies might be necessary for effective prevention of incident ADL dependency among elderly women [40].

Individuals living with chronic health conditions, could not independently perform transportation, to engage in community, social, and civic life; majority had physical disabilities as a limited ability to conduct ADLs independently.”Most persons were independent regarding basic ADLs, for example, self-care activities such as drinking, eating, dressing, and toileting. The activities reported as most dependent were driving, looking after one’s health, and acquisition of goods and services, and assistance was required to perform more complex ADL tasks [46]. The highest percent of unmet ADL needs was for climbing stairs, and the lowest pertained to eating [45].

Dependency in ADL was found more in lower socioeconomic classes compared with higher socioeconomic classes. Compared with males, females are significantly more dependent regarding IADLs. Dependence significantly increased after 80 years of age. The dependence, however, was greater regarding IADL. Education and socioeconomic status have a positive impact on dependency status [47], mainly because learning opportunities can help people develop the skills and confidence to adapt and attempt a healthier ageing process. Elderly people who were working had a lower prevalence of disability for IADLs, which involve more complex activities, and functional disability in general because labour activity implies daily challenges that keep the worker active and contribute to the maintenance of their functional capacity. However, a critical assertion is that elderly individuals might not be working on the grounds of their disability [33].

These elements found to be the most significant: the level of education, the arrangements for living, the number of IADL limiters, the number of diseases, the age of the caregiver, the association of the patient with their caregiver, the size of the household, the burden of care, the link between the service uptake and the welfare expense, and the link between number of IADL limiters and welfare expense [45].

A person who has a mild dependency for care can change into a completely or severely dependent person if the intervention does not take place at the right time. Therefore, it is of utmost importance that care be provided early and that elderly people are monitored to ensure that their progression to complete dependency is slowed down as much as possible [31]. Approximately 93.1% of all disabled people of older age had at least one need that is unmet [31]. The results indicate that early intervention can help decrease the prevalence of reported diseases at more advanced ages [32].

### Psychological problems

Unmet needs and disability can impact both mental and physical health. They can also reduce existing level of QoL and physical health in the context of elderly people. Disability can cause or worsen anxiety and depression, the two main elements that make up psychological distress. Both unmet and met IADL needs are linked to augmented mental distress. To put it simply, being IADL dependent is linked with heightened distress [42]. Caregivers will often only focus on the physical needs of a patient that falls in an older age range. However, activities such as social interaction or a hobby are often ignored, which is a problem. It has been observed that disabled elderly people will normally feel inferior and lose their confidence in regard to talking to other people [31]. Costa, FA, et al. in 2013 [32] showed that depression becomes more prevalent with age. The likelihood of a woman with a disability also showing signs of depression is very high, and the opposite link is also true with an increase in age [32]. Ha and Kim pointed out that as cognitive levels fall, even people that have no past history of mental issues are at risk for behaviour that stems from depression, including destructiveness, violence, agitation [36].

At times, disabled people will not have a choice but to remain in their homes or room. Some even stay in bed for a long period of time to avoid issues. However, not being outdoors for longer periods of time has a detrimental impact on a person’s mental health and can lead to psychological distress for the elderly [31]. Especially regarding disability that impedes the basic activity of urinating or evacuating, this finding is worrying and can lead elderly individuals to experience social isolation, in addition to leading to changes in their self-esteem and self-image, reducing their QoL [33].

Quail, Wolfson, and Lippman in 2011 [44] believed that“there are differences in the severity of psychological distress based upon the type of activity in which a woman is disabled (Personal Activities of Daily Living (PADL) versus IADL) and whether the need for physical assistance is met or unmet. The unmet need to perform an IADL is associated with increased psychological distress over and above the level of distress related to meeting the IADL need. For example, for many women of older generations, cooking is a source of enjoyment. Dependency in meal preparation, regardless of whether the need is met or unmet, may lead to distress because of concerns about not wanting to be a burden, not being able to retain power to decide about meals, or not being able to maintain routine in daily living [42]. Elderly people need to maintain good health, feel happy and comfortable and have more opportunities for social interaction. To improve the QoL of community-dwelling elderly individuals in their communities, supporting them to improve their sense of physical and mental well-being and prevent and reduce their depression and physical pain is required [44].”

### Difficulties in mobility

Malnutrition and cognitive impairment are associated with reduced physical performance and poor muscle strength, leading to disability and reducing the ability to perform basic ADLs [34]. Walking ability has a critical role in the ADL independence of older people [40]. Alva et al. in 2013 [28] described that women who are older and afflicted with sarcopenia find it more difficult to be physically mobile. This is particularly true when the try to climb stairs. The loss of skeletal muscle mass is linked to their decreased physical ability. Elderly women with sarcopenia, compared with those without sarcopenia, are approximately twice as likely to develop difficulties in using stairs [28].

Masoudi Alavi, Safa, and Kalahroudi showed in 2014 [39] that people with fractured hips have poor recovery, and this may impact their mobility, making the issue more permanent [39].

Another aspect was highlighted by Genkai et al. in 2013 [35], who said that occlusal support absence resulted in decreased mobility and physical activity. Muscle strength and a person’s balance are extremely linked with their mobility. Strength in the lower extremities is normally higher in older people in good health as opposed to those who are not. People who have maintained good occlusal support are normally going to have better mobility. In effect, the findings show that occlusal support is essential if one is to preserve the ability to walk. Maintaining this ability has been linked with ADL. The research reviewed shows that occlusal support maintenance is an effective strategy to ensure that one’s walking ability is also maintained [35].

### Poor insight and cognitive function

“Elderly individuals with cognitive impairment may demonstrate minimal impairment in some complex IADLs. For example, tasks often found to be impaired in MCI usually include finances, telephoning, keeping appointments, driving and transportation, shopping, food preparation, and responsibility for medication [30, 37].”

“Cognitive function is a critical factor that affects ADL. Early detection of cognitive disorders is a critical strategy for lowering morbidity. The factors affecting ADL in elderly individuals are faecal incontinence, regularity of exercise, cognitive function, urinary incontinence, and CVA history [36]. Cognitive impairment causes potential problems related to the inability to eat or lack of access to food, hence leading to malnutrition [34].”

Differences in cultural and social background can have an effect on functional assessment. For instance, family structure in many nations consists of not just the immediate family but also the extended family. Therefore, patients with impairments of a cognitive nature typically live with their partners and children, in addition to their siblings and their partners and children. In this situation, it could prove difficult for the caregiver to actually determine how hindered a patient is in regard to performing a given activity. Furthermore, a good number of people might not even know enough about the symptoms of dementia, or its side effects. They think that many symptoms are simply normal for older people, including forgetfulness and ADL decline [30].

### Falls and incidents

Taking a fall could be a marker for “normal” changes that an older person goes through. With age, a person’s strength, gait and vision change. The most significant problem that one faces is the fractures that may result from a fall. Geriatric trauma injuries are also normally the result of a fall [39].“Decreased rapid walking speed increases the risk for falls and therefore increases ADL disability, either from the fracture or post-fall syndrome, in community-dwelling older adults [40].”

Any kind of fracture can have a terrible effect on ADLs; however, hip fractures are the worst. This shows that special follow up in the case of such patients is extremely needed, and this is true for both the postfracture period and the immediate time after it has occurred [41]. Elders can be trained to not get up too quickly in the morning and spend the first couple of minutes sitting on the edge so that they do not cause a fall or topple over [49].

### Wounds and injuries

Trauma, wounds and injuries in older people have a considerable negative impact on their ability and ADL function [39]. The leprosy physical impairment grade is linked with IADL dependence, establishing the requirement for more social support and proper monitoring conducted by a multidisciplinary team. “There is a hierarchy in the process of frailty. First, independence is lost in advanced ADLs, and this loss is followed by a loss of independence in IADLs and, finally, BADLs. The follow-up and rehabilitation of these patients are essential [43].

### Prevalence of undernutrition and dysphagia

“Undernutrition in elderly individuals is a common and important clinical entity that should be diagnosed early; for example, elderly individuals with sarcopenia had a higher prevalence of undernutrition [28]. Additionally, swallowing function, cognitive ability, and nutritional status had direct effects on ADL. Having fewer teeth leads to wearing dentures, but severe cognitive impairment disrupts denture wearing because of problems with, for example, accessing dental care. Chewing difficulties resulting from having fewer teeth and no dentures can lead to dysphagia. Also cognitive impairment can cause potential problems related to the inability to eat or insufficient access to food, leading to malnutrition [34, 35].”

### Communication problems

“Communication is likely to have a significant effect on QoL, considering that it is an ability necessary for instrumental daily life. In particular, stroke patients experience deterioration of social functions due to communication limitations, and when they have difficulty in understanding the meaning of what another party says, or in producing speech, even when they have clear consciousness, it is highly possible that they feel extreme frustration and depression [29].”

## Discussion

Based on the reviewed articles, reducing the problems and improving the conditions of elderly people can be divided into three sections: First, the stage before disability, disease, and their associated problems. Second, the identification and timely diagnosis of disease and problems in elderly people. Finally, the improvement of the conditions of elderly people with disabilities, chronic illnesses, and problems.

**Fig. 3:**
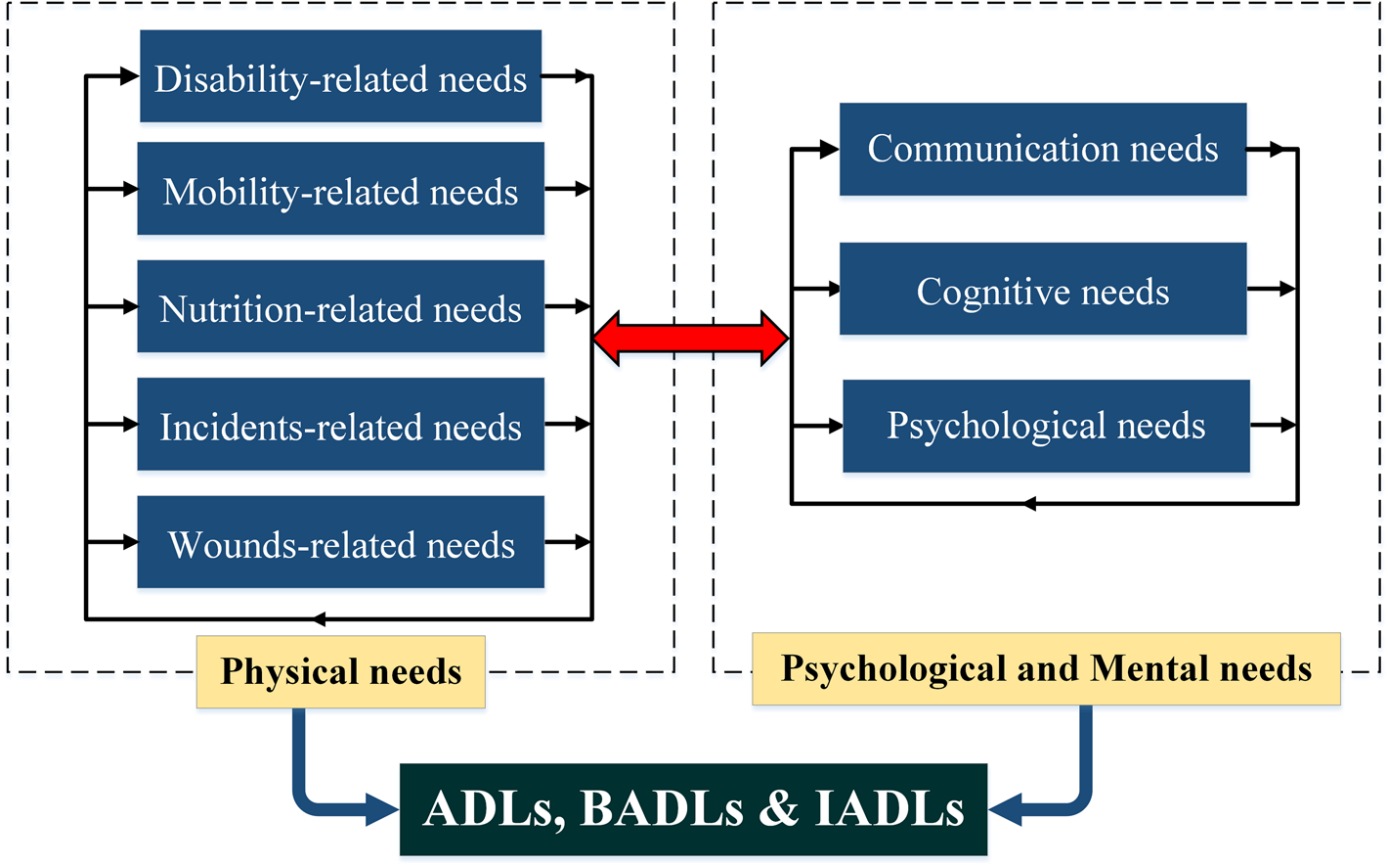
Seniors’ needs based on the distinction made between psychological and physical needs

Given the issues raised in the papers and the categories of the problems experienced by the elderly (as mentioned in the results section), such problems, apart from the developmental time-specific categories, could be further divided into two other types: physical needs and psychological (mental) needs. As the majority of the papers report, most of the age-related disabilities leave a negative psychological impact on old people, along with the limitations that affect the physical aspects of their lives. In Fig. 3, old people’s needs are illustrated based on the distinction between categories of physical needs and psychological needs. As Fig. 3 clarifies, the problems/needs mutually affect each other. Physical problems could intensify psychological ones, while psychological disorder could in turn affect physical well-being. Such problems can reinforce one another’s impact though the relationships they have in dynamic circles. Finally, all of these needs/problems lead to limitations in performing ADLs.

The needs related to mobility, disabilities, and cognition are significant because such dimensions control many everyday life human activities [31, 32]. Such limitations increase the probability of falling, injury and fracture cases, while preventing the individual from attending open urban spaces or from participating in social activities. This negative experience could deeply affect old people psychologically [36, 42]. Therefore, one of the specifically important concerns in this regard is focusing on the solutions that help the elderly to reduce the impacts of disabilities, especially in the case of impaired motor skills [50–52].

Furthermore, cognitive disorders, along with the needs arising from such disorders, represent another important concern [53–55]. Cognitive disorders, besides generating numerous problems for old people in their ADLs, could even result in malnutrition [30, 34], or injures and fractures.

Many studies have mentioned losing independence, pride and confidence as the most serious psychological issues in the elderly. Not being able to do basic activities could isolate old people and undermine their self-esteem [29]. A wide diversity of studies have shown that the ability to do many activities (e.g. procuring food and cooking) brings about a pleasant sense of independence for the elderly; therefore it would be remarkably important to pay attention to needs that can improve an old individual’s independence in addressing his/her personal affairs [34]. Along with these concerns, methods inspiring old people to participate in social activities can prove to be highly important, because depression is one of the consequences of social isolation and limited social interaction [44].

## Conclusion

Chronic diseases can result to ADLs dependency in old age. The major issues that lead to ADLs malfunction in the elderly are disability, psychological disorders, mobility problems, poor cognitive functioning, falling and incidents, wounds and injuries, malnutrition, and communication problems. Within interrelated cycles, old people’s problems are interrelated, and each problem can result in other disorders in such people and finally leave a negative impact on their QoL. On this account, the needs of the elderly are divided into two categories, namely the psychological and the physical. Psychological needs include communication, cognitive and psychological needs. Physical needs are associated with disability, mobility, nutrition, incidents, and wounds. Overall, it would be specifically important to pay attention to methods that can enhance old people’s cognitive abilities, and to methods that can improve their mobility- and disability-related issues; meanwhile establishing conditions inspiring the elderly to take part in social activities could significantly help to improve their life conditions.

This scoping review supports the view on chronic diseases in old age as a complex issue and to prevent the related problems demands multicomponent interventions which includes early recognition of problems leading to disability and ADL dependence. Education and training for health professionals and the general public, can prevent many problems at different levels. Government support and welfare systems should be designed counting complex needs of elderly people. Additionally, the new, upcoming age will be digital and technology based and therefore technology needs to be oriented to solve this problems, which are grouped by this review in eight categories. Satisfying of elderly people will improve their QoL, which should be the ultimate goal.

## Data Availability

The datasets and material used and/or analysed during the current study are available from the corresponding author upon reasonable request.

## Abbreviations

ADLs: Activities of daily living
IADLs: Instrumental activities of daily living
QoL: Quality of life
WHO: World Health Organization
